# Impacto del cálculo del valor seis sigma utilizando la ecuación de Schmidt-Launsbyn vs. la ecuación de Westgard en el programa español EQA tipo I

**DOI:** 10.1515/almed-2024-0209

**Published:** 2025-05-05

**Authors:** Fernando Marqués-García, Elisabeth González-Lao, Xavier Tejedor-Ganduxé, Beatriz Boned, Jorge Díaz-Garzón, Margarida Simón, Jose Vicente García-Lario, Carme Perich, María Pilar Fernández-Fernández, Luisa María Martínez-Sánchez, María Muñoz-Calero, Ricardo González-Tarancón, Pilar Fernández-Calle

**Affiliations:** Departamento de Bioquímica Clínica, Laboratorio Clínico Metropolitana Nord (LCMN), Hospital Universitario Germans Trias i Pujol, Barcelona, España; Sociedad Española de Medicina de Laboratorio (SEQC^ML^), Comisión de Calidad Analítica, Barcelona, España; Consorci Sanitari de Terrassa, Barcelona, España; Departamento de Bioquímica Clínica, Hospital Royo Villanova, Zaragoza, España; Departamento de Medicina de Laboratorio, Hospital Universitario La Paz, Madrid, España; Hospital Universitario San Cecilio, Granada, España; Departamento de Medicina de Laboratorio, Hospital Universitario Central de Asturias, Oviedo, España; Grupo de Bioquímica Clínica Vall d’Hebron Instituto de Investigación, Hospital Universitario Vall d’Hebron, Barcelona, España; Hospital Universitario Reina Sofía, Córdoba, España; Departamento de Bioquímica Clínica, Hospital Universitario Miguel Servet, Zaragoza, España

**Keywords:** seis sigma, Garantía Externa de Calidad (EQA), ecuación de Westgard, ecuación de Schmidt-Launsbyn

## Abstract

**Objetivos:**

La métrica Sigma (SM) mide el rendimiento del proceso a través de los defectos por millón de oportunidad (DPMOs). Tradicionalmente se utiliza la ecuación de Westgard (MW), la cual no realiza una estimación directa de DPMOs. Una alternativa es la transformación Z junto con la ecuación de Schmidt-Launsbyn para el cálculo directo de DPMO. La implementación de SM en los programas de Garantía Externa de Calidad (EQA) es limitada, lo que plantea desafíos para la evaluación de dichos programas. En este trabajo se comparan los valores SM obtenidos por las dos ecuaciones.

**Métodos:**

Se utilizaron datos de un programa EQA de tipo I (SCR-EQA-SEQC^ML^) para estimar el valor sigma (SV) mediante dos métodos: Ecuación de Westgard y transformación Z + ecuación de Schmidt-Launsbyn (S-LM). Se compararon los resultados de ambos métodos.

**Resultados:**

Se incluyeron 949 valores del programa EQA para el cálculo del SV. Seis sigma calculado por MW está subestimado con respecto al valor obtenido por S-LM, tanto con valores atípicos (2,7) como sin ellos (1,9). Esta subestimación está relacionada con el sesgo de tratamiento más que con la imprecisión.

**Conclusiones:**

S-LM gestiona el sesgo, evitando SV negativos a diferencia de MW. Se ve menos afectado por datos extremos, lo que lo hace robusto para el cálculo de SV en programas EQA, garantizando una evaluación precisa de los resultados y la clasificación del rendimiento de los métodos/equipos.

## Introducción

La métrica seis sigma (SM) permite medir la calidad de los procesos de forma objetiva y cuantitativa, determinando los errores producidos en Defectos por Millón de Oportunidad (DPMOs) [1]. El valor seis sigma representa la situación en la que un proceso contiene 3,4 DMPOs [1]. Según el periodo de tiempo utilizado para analizar un proceso, esta estrategia puede evaluarse a largo plazo (calidad máxima 4,5 sigma) o a corto plazo (calidad máxima 6 sigma) [2]. Clásicamente, el valor sigma a corto plazo se calcula como una desviación de 1,5 sigmas sobre el valor sigma a largo plazo [3]. Coskun et al. modifican el concepto de valor sigma a largo plazo indicando que el valor real es 4,65 sigma (Real a largo plazo) y no 4,5 sigma [4].

El valor sigma significa una variabilidad del proceso de 6 desviaciones estándar en torno al valor medio. Lo que implica que un proceso con un nivel de calidad 6 sigma tendrá un número de valores fuera del límite de aceptabilidad establecido (o DPMOs) de 3.4. Un valor sigma de 3 se considera indicativo de una calidad mínima aceptable en los procesos realizados en el laboratorio clínico [3].

Habitualmente, en el laboratorio clínico se utiliza la ecuación propuesta por Westgard para calcular el valor sigma (SV) [3]. Mediante esta ecuación, se establece una relación lineal entre el sesgo y el objetivo analítico establecido por error total (ET), e inversamente proporcional para la imprecisión; y se estiman indirectamente las DMPOs. El objetivo principal de SM es calcular directamente el número de DMPOs en los procesos a evaluar. Este aspecto nos ha hecho replantearnos la necesidad de valorar cuál es la estrategia más adecuada para la evaluación de la SM en los procesos analíticos de laboratorio [4]. Como alternativa, se propone la estimación directa de DMPOs utilizando la estrategia de transformación Z como patrón oro [5] para el cálculo de la métrica sigma. Para complementar el cálculo, el valor de DMPOs se transforma en un cálculo de sigma mediante la ecuación de Schmidt-Launsbyn (S-L) [6], [7].

Actualmente, el grado de implantación de la estrategia SM en los programas de Garantía Externa de Calidad (EQA) es limitado [8]. La incorporación de SM a los programas EQA representa un aspecto de mejora para evaluación del desempeño de estos programas. Estos programas representan una herramienta esencial para conocer el desempeño analítico de cada laboratorio, así como para permitir el desarrollo de propuestas de mejora de métodos y equipos. La capacidad de evaluar el rendimiento de los laboratorios depende del diseño del programa EQA, así se han descrito cinco categorías [9]. Los programas más recomendados son aquellos que utilizan materiales de control conmutables, con valores asignados por métodos o materiales de referencia, y en los que se analizan réplicas de las muestras (Categoría 1). La Sociedad Española de Medicina de Laboratorio (SEQC^ML^) dispone de un programa EQA de Categoría 1 (SCR-EQA-SEQC^ML^) para 17 magnitudes biológicas, que permite realizar una evaluación más completa de estos métodos. Por último, es necesario disponer de procedimientos robustos para el cálculo de SM que permitan una adecuada evaluación de los instrumentos y métodos participantes en los programas EQA.

El objetivo principal de este estudio fue calcular el SV utilizando diferentes estrategias: la ecuación de Westgard y la estrategia basada en la transformación Z con aplicación de la ecuación S-L, con el fin de establecer cuál de ellas ofrece mejores prestaciones en la evaluación de los datos del programa SCR-EQA-SEQC^ML^.

## Materiales y métodos

### Materiales

En este estudio se emplearon los resultados obtenidos en el programa SCR-EQA-SEQC^ML^. Este programa utiliza material de control conmutable a partir de suero humano fresco con valor asignado por métodos de referencia (categoría 1) [10], [11].

Los materiales de control utilizados para este estudio se describen detalladamente en el artículo de Ricos et al. [10]. El material de control se adquirió a la Stichting Kwaliteitsbewaking Medische Laboratorium Diagnostiek (SKML) y se preparó en el laboratorio MCA (Hospital Reina Beatriz, Winterswijk, Países Bajos). Se enviaron seis viales de material de control a diferentes concentraciones (6 niveles) a cada participante en el programa en un único envío a −80 °C. Cada vial suministrado se midió por duplicado durante 6 días consecutivos y los resultados obtenidos se recogieron en la página web del programa SCR-EQA-SEQC^ML^.

Los resultados se incluyeron en el estudio cuando participaban al menos 5 laboratorios en el grupo homogeneo. Para cada una de las magnitudes biológicas incluidas en el programa se calculó la media y el coeficiente de variación (CV, %) correspondiente a cada método-instrumento y nivel de concentración. Las magnitudes biológicas incluidas en el programa SCR-EQA-SEQC^ML^ se indican en la Tabla 1. La tabla incluye tanto la especificación de calidad establecida por el programa EQA en base a la variación biológica [12], como la concentración a la que se establece el nivel de decisión clínica [13]. Este trabajo considera los resultados de los laboratorios participantes en un EQA no de forma individual, sino colectivamente en grupos homogéneos, con el mismo método analítico-instrumento y trazabilidad del calibrador.

**Tabla 1:** Especificaciones de calidad para el error total (ET) fijadas por el programa SCR-EQA-SEQC^ML^ y niveles de decisión clínica para las magnitudes incluidas en el estudio.

Magnitud biológica	Especificaciones del programa de calidad/ET EQA	Nivel de decisión clínica	
α-Amilasa	VB óptima/6.5 %	120	U/L
ALP	VB deseable/10.4 %	150	U/L
ALT	VB óptima/9.2 %	60	U/L
AST	VB óptima/6.1 %	60	U/L
Bilirrubina total	VB óptima/12.3 %	42.7	µmol/L
Calcio	VB mínima/3.3 %	2.7	mmol/L
CK	VB óptima/10.3 %	240	U/L
Cloro	VB mínima/1.9 %	112	mmol/L
Creatinina	VB deseable/7.8 %	141.4	µmol/L
GGT	VB óptima/9.1 %	50	U/L
Glucosa	VB deseable/6.2 %	6.6	mmol/L
LDH	VB óptima/3.4 %	300	U/L
Magnesio	VB deseable/3.8 %	1	mmol/L
Potasio	VB deseable/4.9 %	5.8	mmol/L
Proteínas totales	VB mínima/5.1 %	60	g/L
Urato	VB óptima/6.3 %	475.8	µmol/L
Sodio	VB mínima/1.1 %	135	mmol/L

TE, error total; EQA, garantía externa de calidad; VB, variación biológica; AST, aspartato aminotransferasa; ALT, alanina aminotransferasa; ALP, fosfatasa alcalina; GGT, γ-glutamiltransferasa; CK, creatina kinasa; LDH, lactato deshidrogenasa. Óptima, deseable y mínima se refiere al nivel de especificación por variación biológica.

### Estrategias y análisis estadístico

En este estudio, el SV se calculó siguiendo dos estrategias diferentes: utilizando la ecuación propuesta por Westgard (MW) [14], [15], y la estrategia de transformación Z seguida de la aplicación de S-L (S-LM). Los cálculos se realizaron para cada grupo instrumento-método y para cada una de las magnitudes biológicas estudiadas.

### Estrategia Westgard para el cálculo de seis sigma

Para el cálculo del SV mediante la ecuación de Westgard (MW) se utilizó el valor del programa SCR-EQA-SEQC^ML^ para la imprecisión (CV, %) y el sesgo (ES, %). Para cada magnitud y nivel de control, la imprecisión se determinó como el porcentaje de dispersión de los datos, mientras que el sesgo se estableció como la diferencia del valor medio de cada concentración de control, con respecto al valor obtenido por el método de referencia. La especificación para el error total (ET, %) del programa SCR-EQA-SEQC^ML^ se estableció como el límite de aceptación de la distribución de los datos (Tabla 1). El SV se calculó mediante la siguiente fórmula para cada grupo homogeneo y nivel de concentración:

$$\text{SV}=\left(\text{ET}-\left|\text{ES}\right|/\text{CV}\right)$$

Todos los parámetros de la ecuación se expresan en porcentaje (%). El SV calculado se transformó en DPMOs mediante tablas de conversión (https://sixsigmastudyguide.com/process-performance-metrics/).

### Estrategia de transformación Z y ecuación de Schmidt-Launsbyn para el cálculo de seis sigma

Para el cálculo del SV mediante la estrategia de transformación Z seguida de la aplicación de la ecuación de Schmidt-Launsbyn (S-LM), se utilizaron los valores SD obtenidos para cada magnitud y nivel de control, y ET fijado como especificación por el programa SCR-EQA-SEQC^ML^ (Tabla 1). En primer lugar, se calcularon los límites establecidos para el cumplimiento de la especificación:

Límite superior=Valor de referencia+ET

Límite inferior=Valor de referencia−ET

El valor de referencia corresponde al valor objetivo del material de control obtenido por un método de referencia (programa EQA tipo 1). La distribución normal se caracteriza por tener una media de 0 (µ) y una imprecisión de 1 (σ). Para normalizar la distribución de los datos, se utilizó la herramienta estadística de transformación Z [16], que muestra cuánto puede variar el resultado con respecto al objetivo óptimo antes de producir un valor fuera de especificaciones. Del mismo modo, se calcularon las puntuaciones Z de los dos límites de la distribución obtenidos anteriormente del grupo homogeneo y nivel de concentración. Se aplicaron las siguientes fórmulas:

$$\text{Puntuación}\;Z\text{ superior}=\left(\text{Lí}\text{mite}\text{ superior}-\text{Valor notificado por el laboratorio}\right)/\text{SD del grupo homogeneo}$$

$$\text{Puntuación}\;Z\text{ inferior}=\left(\text{Lí}\text{mite}\text{ inferior}-\text{Valor notificado por el laboratorio}\right)/\text{SD del grupo homogeneo}$$

La diferencia entre los valores de la distribución normal estándar de la puntuación Z superior e inferior define el área bajo la curva (AUC) [3] contenida dentro de las especificaciones establecidas. La diferencia entre el AUC definido por las especificaciones y el AUC calculado representa los defectos que se están produciendo en el proceso. El valor de esta área se multiplica por 10^6^, obteniéndose el número de DPMOs. Por último, con el valor de DMPOs obtenido, se calcula el SV aplicando la ecuación S-L [6], [7]:

$$S-L=0.8406+(\sqrt{\left(\left(29.37-2.221*\mathrm{Ln}\left(\text{DMPOs}\right)\right)\right)}$$

Para el análisis de los valores atípicos se utilizó el método de Tukey [17].

Para el análisis estadístico y la generación de gráficos se utilizó el programa Excel (Microsoft Corporation^®^, Redmon, Washington, EE.UU.).

## Resultados

**Figura 1:**
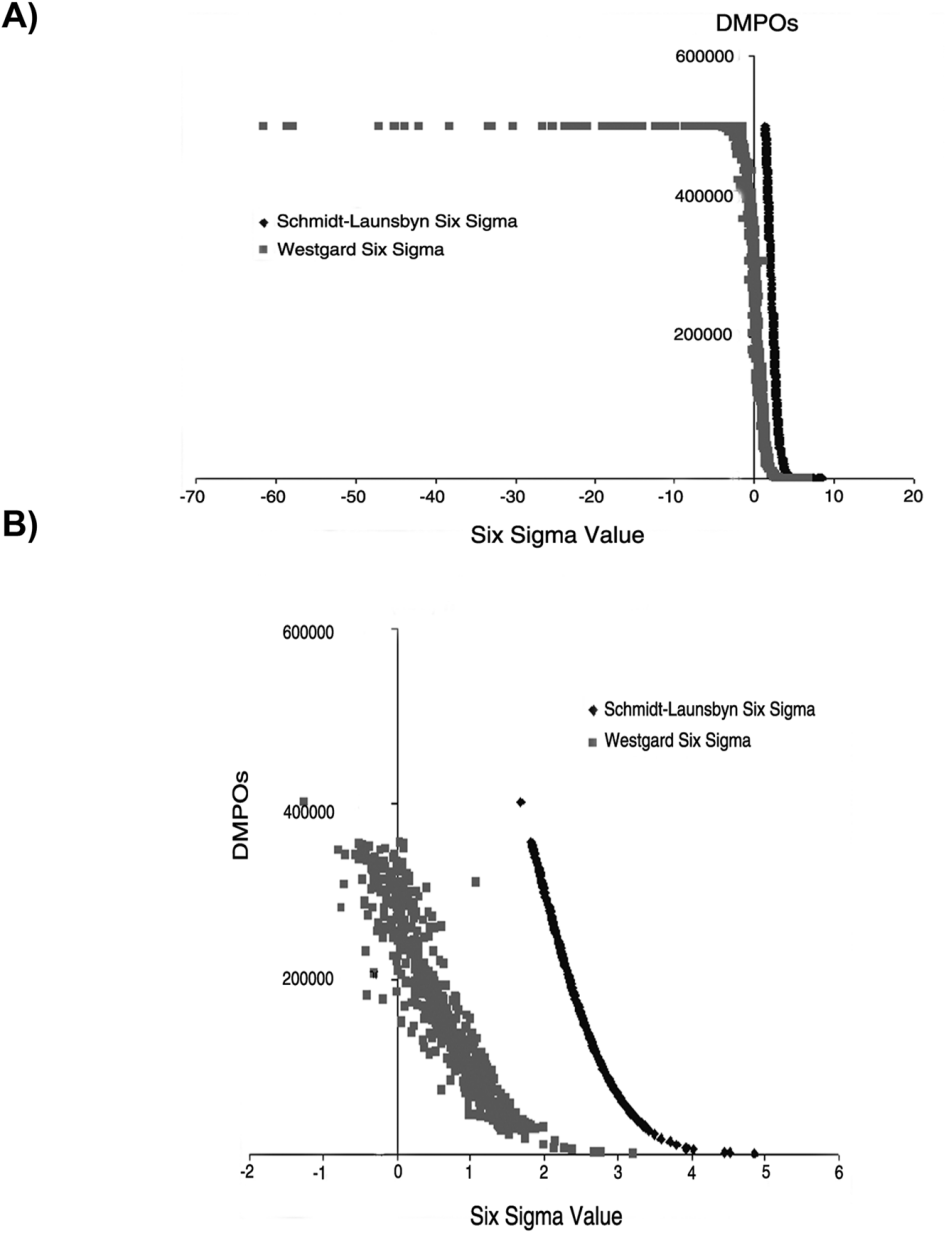
Relación entre los valores de seis sigma y el número de DMPO por los métodos MW y S-LM. Se representan los valores de seis sigma (MW y S-LM), ordenados de mayor a menor (de izquierda a derecha) (A), y un detalle de (A) entre 5 y -1 valores de seis sigma (B). El eje de abscisas representa el valor seis sigma y el eje de ordenadas representa el número de DMPOs. DMPOs: defectos por millón de oportunidad, S-LM: Método de transformación Z-Schmidt-Launsbyn, MW: Método Westgard. En la Figura se incluyen los datos de todas las magnitudes del programa.

Para los cálculos de SM se incluyeron 949 resultados (todos los: niveles de concentración, del grupo homogeneo con más de 5 participantes y magnitudes biológicas del programa). Los SV obtenidos se agruparon en valores calculados mediante la MW y la estrategia S-LM. Estos valores se representaron en gráficos en los que se relacionaba el número de DPMOs con el SV obtenido (Figura 1 y Figura Suplementaria 1). La Figura 1 muestra las diferencias entre los SV calculados por MW y S-LM, desde un valor de seis sigma hasta 0. Utilizando la ecuación MW se obtienen valores sigma negativos (hasta −60) a partir de 2 × 10^5^ DPMOs (Figura 1A and B); en cambio utilizando la ecuación S-LM no se obtienen valores negativos ni siquiera con DPMOs superiores a 2 × 10^5^. Se seleccionó un rango desde el valor 400 hasta 1,100 ordenando de mayor a menor valor de seis sigma (Figura 1B), y se graficaron los resultados. En esta zona, se puede observar cómo la relación entre DPMOs y el SV es única y directamente proporcional utilizando la estrategia S-LM (Figura 1B).

Por otro lado, utilizando el MW se obtiene una nube de puntos en la que se obtienen diferentes SV para el mismo número de DMPOs (Figura 1B) y viceversa. La Figura Suplementaria 1 representa la evolución de la curva representada en la Figura 1A, pero añadiendo 100 valores a cada gráfico. A partir de los SV, tanto los calculados por MW como por S-LM, se obtuvo el SV medio de todos los valores del programa SCR-EQA-SEQC^ML^ sin eliminar los valores atípicos, o con la eliminación de los valores atípicos haciendo que el SV medio obtenido por MW aumente, mientras que el valor obtenido por S-LM no se ve afectado. No se observaron diferencias entre los dos grupos sin valores atípicos (Tabla 2).

**Tabla 2:** Valores promedio de Seis Sigma considerando los datos totales del programa EQA a nivel de decisión clínica.

Datos seleccionados	S-L	W	(S-L)-W	Imprecisión, %	Sesgo, %
Nivel de decisión clínica con valores atípicos	3.20	0.52	2.67	3.49	7.36
Nivel de decisión clínica sin valores atípicos	3.23	1.33	1.89	3.12	3.90
Todos los datos EQA con valores atípicos	3.06	0.31	2.75	3.77	8.33
Todos los datos EQA sin valores atípicos	3.10	1.14	1.96	3.23	4.37

S-L, Schmidt-Launsbyn; (S-L)-W, método Schmidt-Launsbyn menos método Westgard; EQA, garantía externa de calidad; W, Westgard.

Para el valor medio del SV por S-LM, se observaron diferencias menores entre los grupos con y sin valores atípicos (3.06 a 3.10) (Tabla 2). Por otra parte, utilizando la misma comparación con los datos obtenidos por MW, se observan diferencias superiores de 0.31 a 1.14. En los dos grupos estudiados, la eliminación de los valores atípicos afecta al valor del sesgo (reduciéndolo), pero no a la imprecisión (Tabla 2/Figura 1A).

La relación Schmidt-Launsbyn-Westgard ((S-L)-W) permite evaluar la diferencia entre los SV obtenidos mediante las dos estrategias. La diferencia en el SV calculado por (S-L)-W oscila aproximadamente entre 2.7 y 1.9 considerando la población de datos con o sin valores atípicos, respectivamente (Tabla 2). Así, se puede comprobar como MW subestima el SV, disminuyendo esta subestimación si excluimos los valores extremos.

Para observar la tendencia de los SV individuales, los representamos considerando todas las magnitudes biológicas incluidas en el programa SCR-EQA-SEQC^ML^ a nivel de decisión clínica (Figura Suplementaria 2). Se consideró el S-LM como valor para ordenar los resultados de menor a mayor, comprobando la diferencia proporcional (subestimación) en el SM MW con respecto al valor de S-LM (Figura Suplementaria 2). Cuando los SV MW son negativos, esta diferencia entre las dos formas de cálculo se hace exponencial. En cambio, los SV positivos presentan una diferencia constante entre las dos formas de cálculo. Los datos se han analizado eliminando los valores atípicos. Si se incluyen los valores atípicos, la relación exponencial provocada por los SV negativos WM se agudiza, lo que dificulta la visualización de los resultados en la zona lineal de la relación (Figura Suplementaria 3).

**Figura 2:**
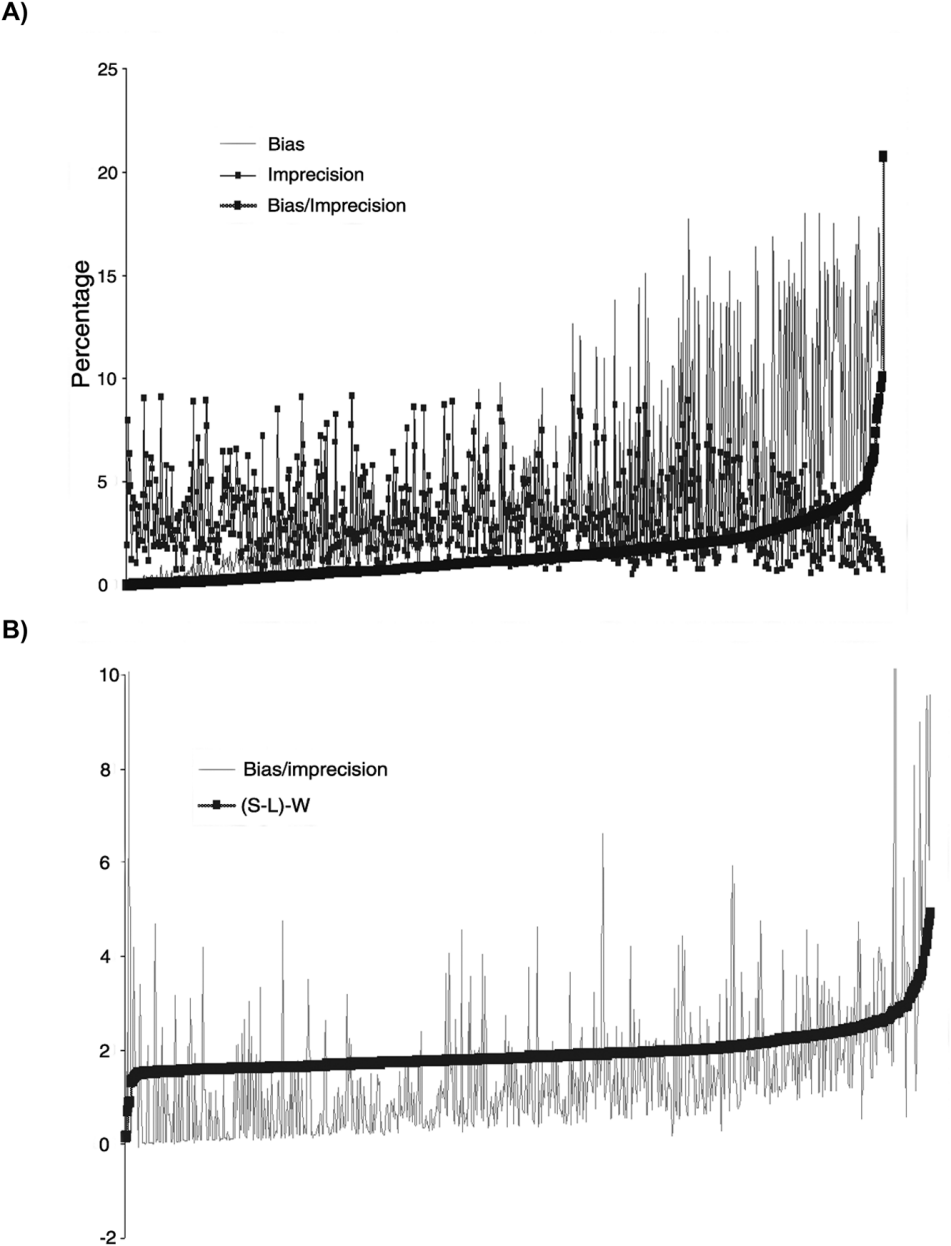
Influencia del sesgo e imprecisión del programa EQA en la estimación del valor promedio de seis sigma para las dos estrategias utilizadas. Las figuras representan todos los datos de EQA ordenados según el cociente de sesgo/imprecisión (A) y según el valor de la diferencia (S-L)-W (B) del valor menor al mayor. Los dos gráficos muestran los datos sin valores atípicos. Los datos se ordenan de izquierda a derecha de menos a más valor de bias/imprecisión (A) y (S-L)-W (B).

La relación entre sesgo e imprecisión de los resultados emitidos obtenida por los grupos homogéneos de laboratorios participantes en el programa EQA, eliminando los valores atípicos, se representa en la Figura 2. Si ordenamos estos datos de menor a mayor relación sesgo/imprecisión, ésta aumenta principalmente a expensas del incremento del valor del sesgo. La imprecisión permanece constante e incluso disminuye ligeramente en los valores más altos de la relación sesgo/imprecisión (Figura 2A). Del mismo modo, un aumento del valor de la diferencia (S-L)-W aumenta el valor de la relación sesgo/imprecisión, lo que indica el efecto que tiene el sesgo sobre la estrategia de cálculo de SV mediante MW (Figura 2B). El efecto sobre el sesgo aumenta si tenemos en cuenta los valores atípicos (Figura Suplementaria 4).

Finalmente, tomando el SV obtenido por S-LM, las magnitudes biológicas se agruparon en aquellos con un nivel superior a 4 sigma (3 magnitudes biológicas), entre 4-3 (7 magnitudes biológicas) e inferior a 3 (8 magnitudes biológicas) (Tabla 3).

**Tabla 3:** Magnitudes del programa SCR-EQA-SEQC^ML^ agrupadas según el valor sigma calculado mediante S-LM.

Valor Sigma	Magnitudes biológicas
Mayor de 4	CK
	Potasio
	Creatinina enzimática
Entre 4 y 3	Proteínas totales
	ALP
	GGT
	Creatinina Jaffé
	Urato
	Glucosa
	Bilirrubina total
Menor de 3	Calcio
	ALT
	α-Amilasa
	Cloro
	Magnesio
	LDH
	AST
	Sodio

EQA, garantía externa de calidad; AST, aspartato aminotransferasa; ALT, alanina aminotransferasa; ALP, fosfatasa alcalina; GGT, γ-glutamiltransferasa; CK, creatina kinasa; LDH, lactato deshidrogenasa.

## Discusión

### Comparación entre las dos estrategias estudiadas

Basándonos en la literatura, en el laboratorio clínico se han seguido dos estrategias para el cálculo del SV. Clásicamente, según el enfoque de Westgard (MW), el SV se ha estimado de forma indirecta, estableciendo una relación proporcional entre los errores analíticos: error total (ET), sesgo e imprecisión [18]. Alternativamente, en los últimos años, se ha propuesto el cálculo de SM basado en la determinación directa de DPMOs [19]. Esta estrategia se centra en la transformación de los datos en una distribución normal, mediante un proceso de transformación Z, que se ha descrito como la metodología con mejores resultados para calcular SM [5]. La relación entre las DMPOs y el SV no es lineal sino exponencial [20], [21]. En este trabajo, introducimos el uso de la fórmula S-L para transformar las DMPOs en un valor sigma siguiendo la relación logarítmica que presentan estos dos valores (S-LM). Utilizando esta ecuación, obtenemos un SV único para cada valor de DPMO y directamente proporcional. Por otro lado, MW estima el SV indirectamente sin el conocimiento de las DMPOs, lo que significa que diferentes combinaciones de sesgo e imprecisión generan un SV igual (Figura 1).

Además, a medida que aumenta el sesgo analítico, tienden a aparecer SV aberrantes (negativos) a partir de un valor aproximado de 2 × 10^5^ DMPOs. Cuando el valor del sesgo es superior a los límites establecidos de la distribución (por ejemplo, especificación de calidad por ET del programa EQA) comienzan a aparecer los valores negativos descritos. Esto está relacionado con el tratamiento lineal de los errores (principalmente el sesgo) cuando se aplica la ecuación de Westgard [3], mientras que la relación entre SV y DMPOs sigue una distribución normal [22]. No hay variación en los comportamientos descritos si se analizan todos los datos o si nos centramos en los valores próximos al nivel de decisión clínica. Debido a los problemas asociados a la medición del SV mediante la ecuación de Westgard, se eliminaron los valores atípicos de la distribución de datos (los valores de sesgo más alto), lo que condujo a una reducción significativa de los SV negativos, aunque no por completo. Por otra parte, la imprecisión apenas sufrió variaciones en función de la estrategia de cálculo de SM (Westgard o S-L) o del manejo de los valores atípicos (presencia o ausencia).

Del mismo modo, el SV medio para el programa SCR-EQA-SEQC^ML^ calculado mediante S-LM no varió significativamente cuando se mantuvieron o eliminaron los valores atípicos, considerando toda la población de datos (3.06 frente a 3.10) o sólo los correspondientes al nivel de decisión clínica (3.20 frente a 3.23). Sin embargo, el SV MW mostró una mayor variación en función de si se incluían o no los valores atípicos, tanto en el nivel de decisión clínica (0.52 frente a 1.33) como al considerar todos los datos del programa SCR-EQA-SEQC^ML^ (0.31 frente a 1.14). Por otro lado, la relación sesgo/imprecisión disminuyó al eliminar los valores extremos debido a la disminución del sesgo. Por lo tanto, se comprueba que el SV S-LM no se ve afectado por los valores extremos, al contrario de lo que ocurre con los valores calculados por Westgard, que se ven influidos por el valor del sesgo. La forma en que cada estrategia tiene en cuenta el sesgo contribuye fundamentalmente a las diferencias observadas en el SV. Si relacionamos los SV obtenidos por las estrategias S-LM y MW, se observa una diferencia constante entre ambos valores, que muestran una subestimación del SV por parte de la MW que oscila entre 2.7 con valores atípicos, y 1.9 tras eliminar los valores atípicos. Las diferencias en el SV entre ambas estrategias ((S-L)-W) aumentan a medida que aumenta el valor del sesgo (aumento relación sesgo/imprecisión) (Figura 2A y B).

El impacto de la subestimación del valor sigma al aplicar el MW afecta al nivel de cumplimiento de los objetivos de calidad analítica del programa SCR-EQA-SEQC^ML^. El MW está muy influido por valores de sesgo elevados, lo que hace que se obtengan SV negativos. En cambio, con el S-LM evitamos este problema tratando estadísticamente el valor del sesgo de forma más adecuada. Una gestión adecuada del sesgo mejora la evaluación del rendimiento de los diferentes métodos e instrumentos participantes, y del programa EQA en su conjunto.

### Desempeño del programa EQA

Un programa EQA sirve para evaluar las prestaciones analíticas. Utilizando la estrategia MW se obtienen valores sigma inadecuados que oscilan, entre 1.33 con valores atípicos y 1.14 sin valores atípicos, ambos a nivel de decisión clínica. En cambio, con la estrategia S-LM los valores fueron de 3.23 y 3.10 respectivamente. De esta manera, el desempeño global del programa superaría el límite aceptado en el Laboratorio Clínico para SV establecido en 3 sigma. Este valor nos da una perspectiva global del programa, así conocer el valor sigma para cada magnitud nos ayuda a implementar acciones sobre aquellos magnitudes biológicas con bajo rendimiento. Sólo 3 magnitudes biológicas presentan valores superiores a 4 sigma (CK, potasio y creatinina enzimática), 7 magnitudes biológicas entre 4-3 sigma y 8 por debajo de 3 sigma. Como 3 sigma es el mínimo, en magnitudes biológicas con SV inferior a 3, se deben incrementar los esfuerzos de los Laboratorios Clínicos y/o empresas de diagnóstico *in vitro* (IVD) para mejorar las prestaciones. Por otro lado, las magnitudes biológicas entre 4-3 sigma tienen que mejorarse para llegar al nivel 4 sigma. Destacar la creatinina, el uso de un método más preciso como es la creatinina enzimática presenta un mejor valor sigma (mayor a 4) que el método de Jaffé (entre 4-3 sigma). Este es un claro ejemplo de cómo los laboratorios clínicos deben tender a utilizar e implementar los métodos analíticos disponibles que presenten el mejor rendimiento posible. La estrategia de cálculo de SM influye significativamente en el valor obtenido y en los objetivos a cumplir dentro del programa EQA.

La métrica seis sigma se presenta como una herramienta con un alto potencial de aplicación dentro de los programas EQA ya que actúa como un elemento que permite tener una visión del desempeño (imprecisión y sesgo) de los métodos e instrumentos del laboratorio integrado en un solo indicador. Además, SM permite conseguir un mayor grado de armonización entre los diferentes programas EQA, disponiendo de un parámetro de intercomparación común entre todos ellos.

Actualmente, el grado de implementación de la métrica seis sigma dentro de los programas EQA es limitado. El programa holandés SKML lo incorpora en el informe de evaluación de los resultados, pero utilizando la estrategia Westgard notablemente limitada, como se evidencia en los resultados de este estudio. La Tabla 3 presenta la agrupación de métodos, por magnitud biológica, estudiados en el programa SCR-EQA-SEQC^ML^ en base a su valor seis sigma. La evaluación del rendimiento analítico presente en esta tabla es acumulativa para cada magnitud biológica. Por lo tanto, para cada magnitud biológica, es necesario evaluar cada programa EQA para los equipos individualmente. Este estudio será abordado más adelante, ya que el objetivo del artículo actual es evaluar las estrategias para calcular seis sigma. En general, para las magnitudes biológicas con valor sigma bajo es necesario incidir en la mejora de los métodos por la IVD, lo que nos permitirá alcanzar los rendimientos analíticos establecidos en base a la variación biológica. Por ejemplo, en el caso del sodio, utilizar potenciometría directa, en lugar de potenciometría indirecta, permitiría mejorar el valor seis sigma.

Como conclusión, el uso de la estrategia S-LM se ajusta a los principios fundamentales que se deben cumplir para el cálculo de SM, como asegurar la distribución normal de la población de datos, y resuelve las limitaciones presentes en la estrategia que utiliza la metodología MW. Por lo tanto, el enfoque S-LM nos permite calcular el valor de los DMPO directamente para a continuación calcular el SV, a diferencia de la estrategia basada en la ecuación de Westgard. A través del S-LM, cada valor de DPMO corresponde a un único SV. S-LM nos permite obtener valores más robustos de SV ya que su valor a través de S-LM no se ve afectado por valores extremos. A diferencia del MW, S-LM realiza un tratamiento no lineal del sesgo. Este tratamiento del sesgo hace que se disminuya la influencia en el SV. Este trabajo destaca la importancia del sesgo en el SV obtenido y la selección adecuada de la estrategia más apropiada para calcular SM. Una estrategia robusta para calcular SM podría facilitar su aplicación dentro de los programas EQA, permitiendo una evaluación objetiva del proceso analítico de los diferentes participantes.

## Supplementary Material

Supplementary Material

## Material Suplementario

Este artículo incluye material suplementario https://doi.org/10.1515/almed-2025-0091.
