# Supplementary material for: Impacto del cálculo del valor seis sigma utilizando la ecuación de Schmidt-Launsbyn vs. la ecuación de Westgard en el programa español EQA tipo I
Source: Adv Lab Med. 2025 May 5;6(3):336–44. [Article in Spanish] doi: 10.1515/almed-2024-0209 (PMC12446908; doi:10.1515/almed-2024-0209)
Supplement: Supplementary file 1 — Supplementary Material [file j_almed-2024-0209_suppl_001.doc]

**Figuras Suplementarias**

**Figura Suplementaria 1.** Evolución de las curvas del valor seis sigma calculado por las dos ecuaciones propuestas a medida que se van añadiendo de 100 en 100 los datos del programa EQA. Estos gráficos se han hecho ordenando los datos por el número de defectos por millón de oportunidad (DPMO).

**
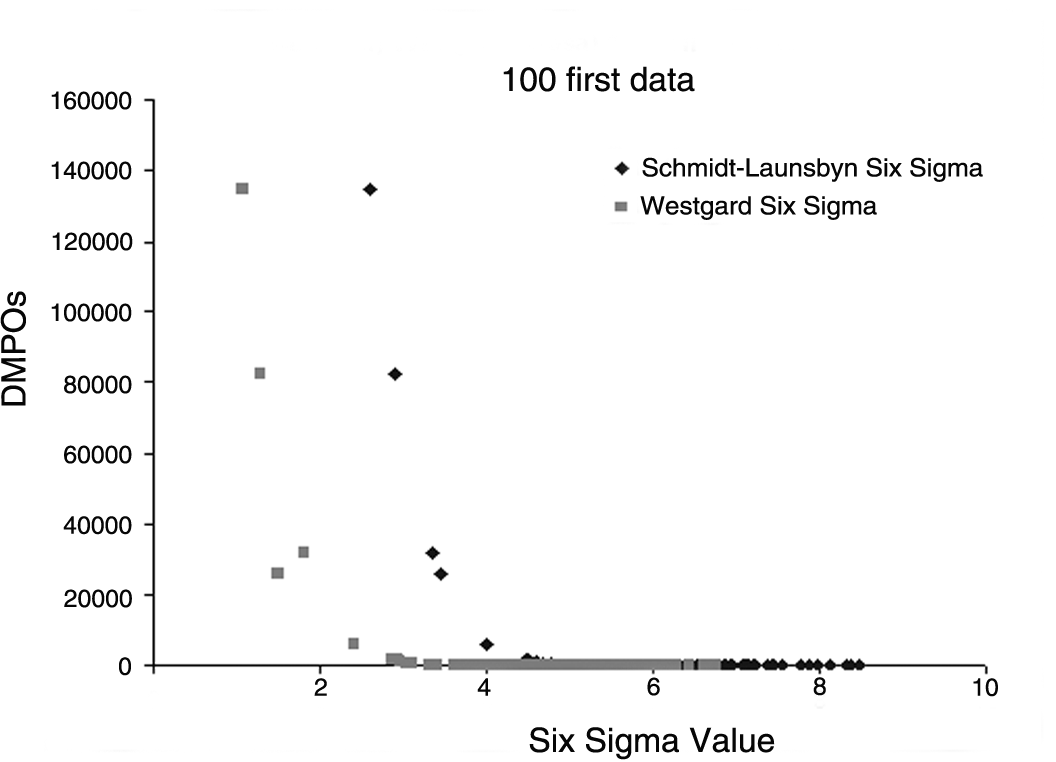

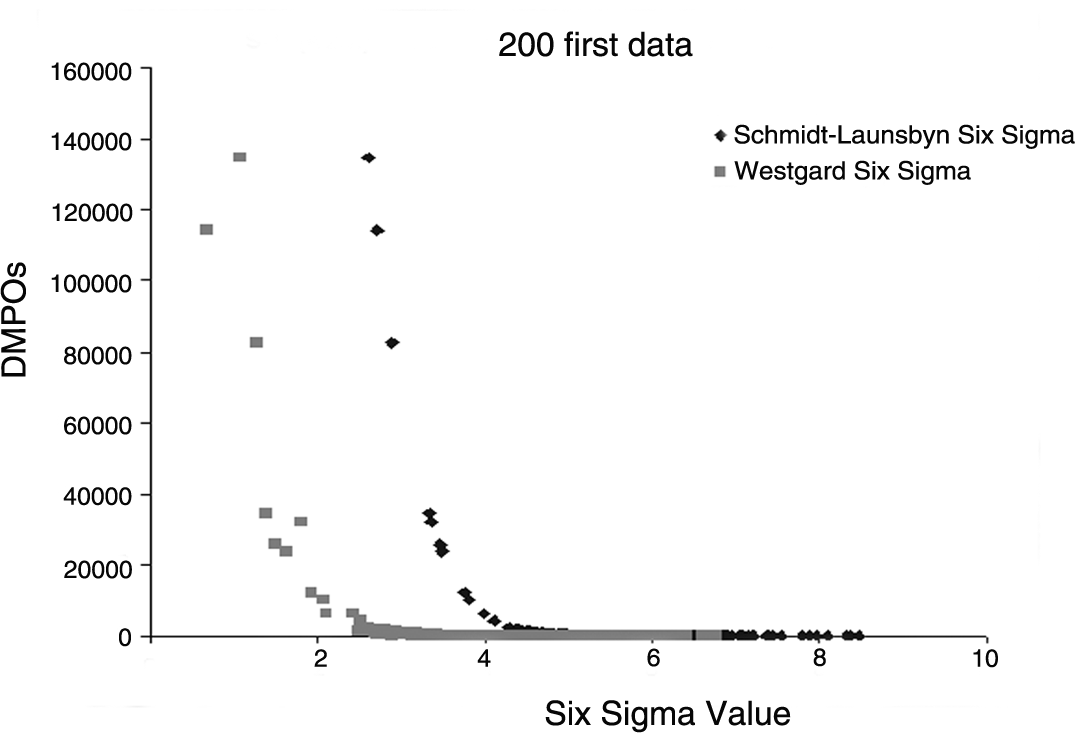
**


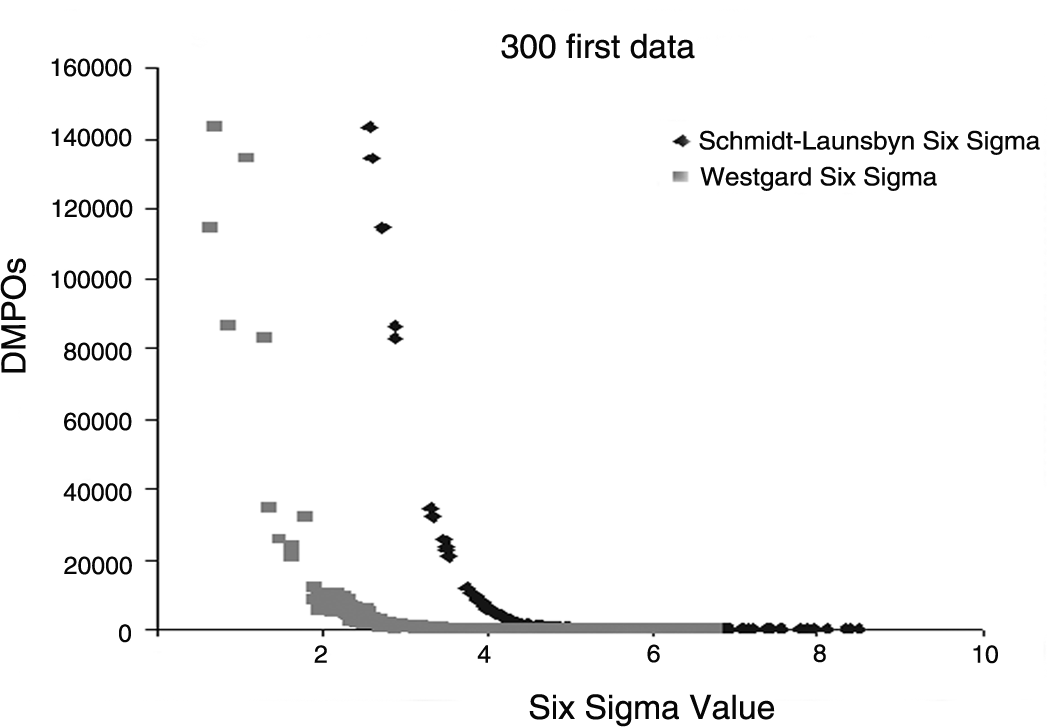

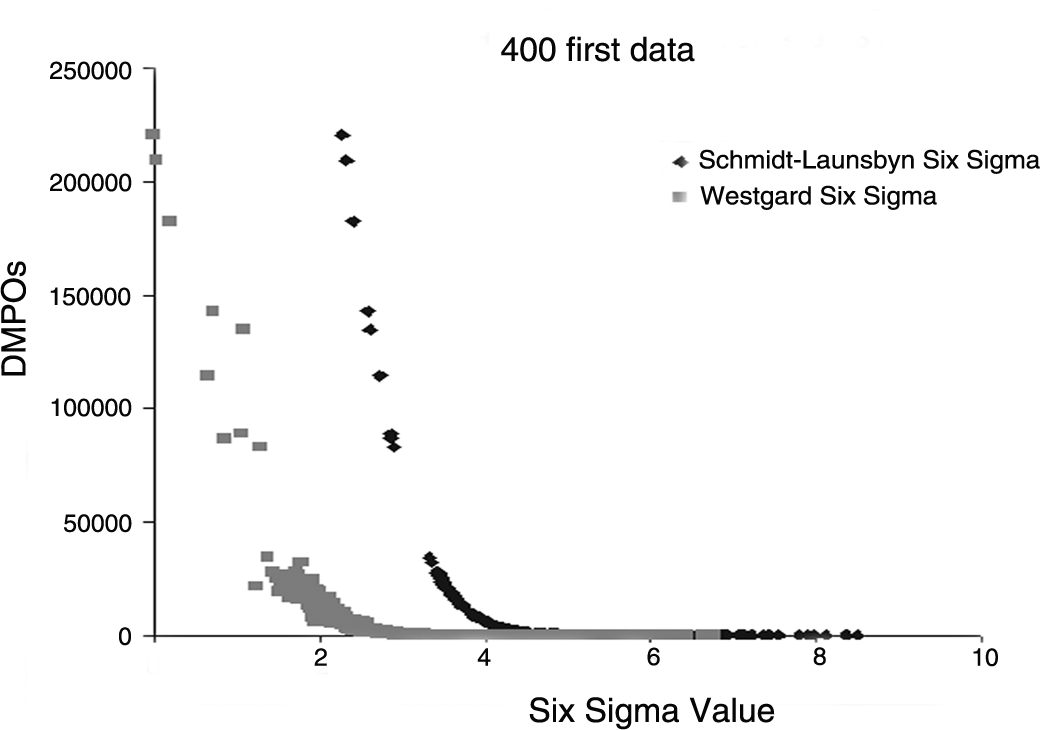


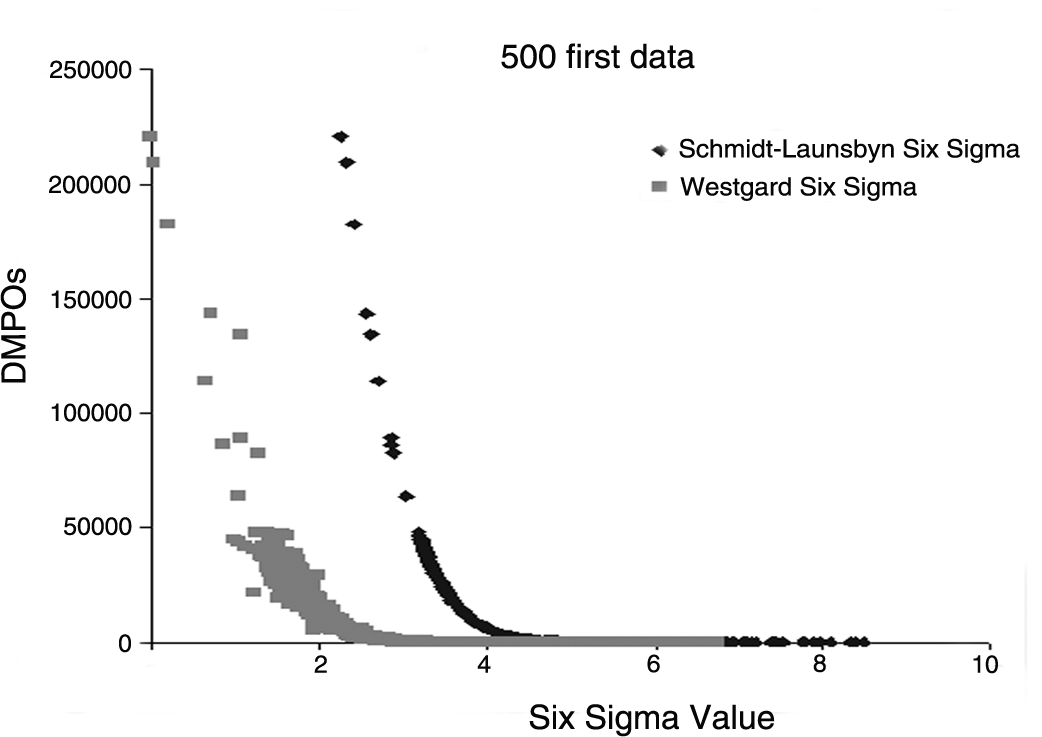


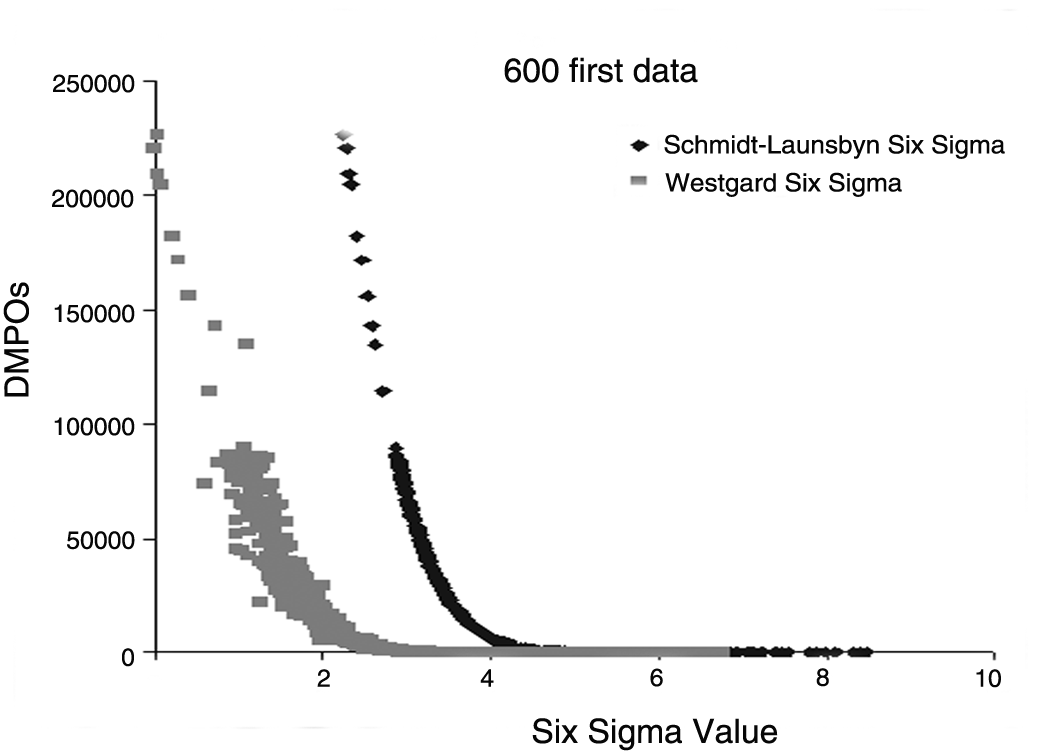


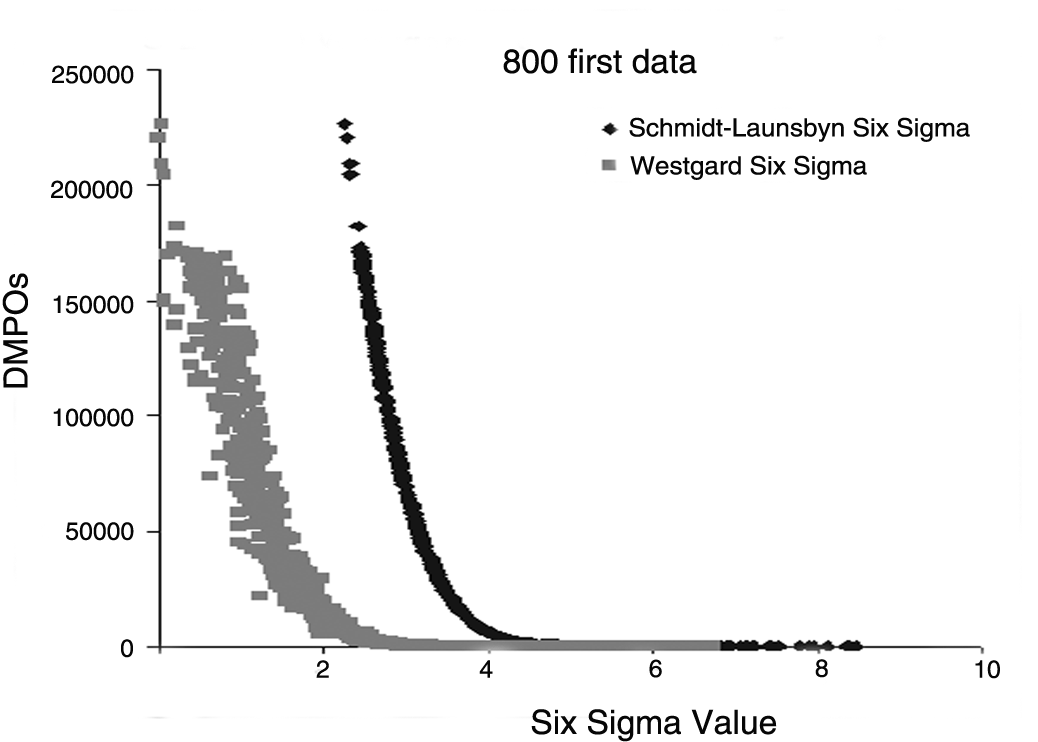


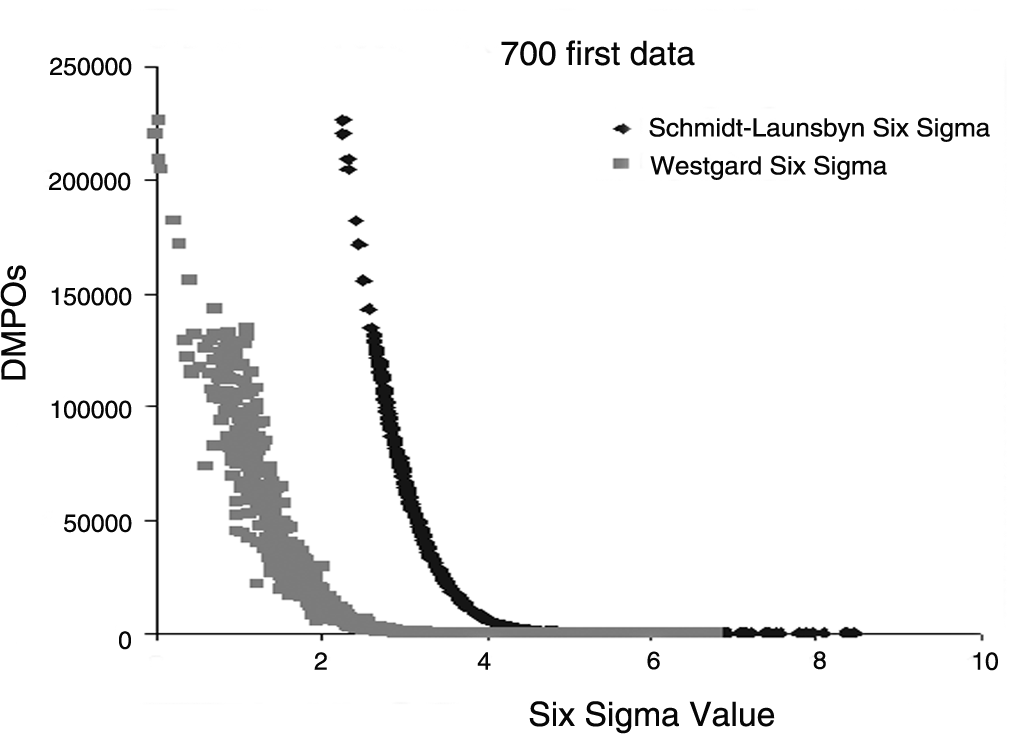


**
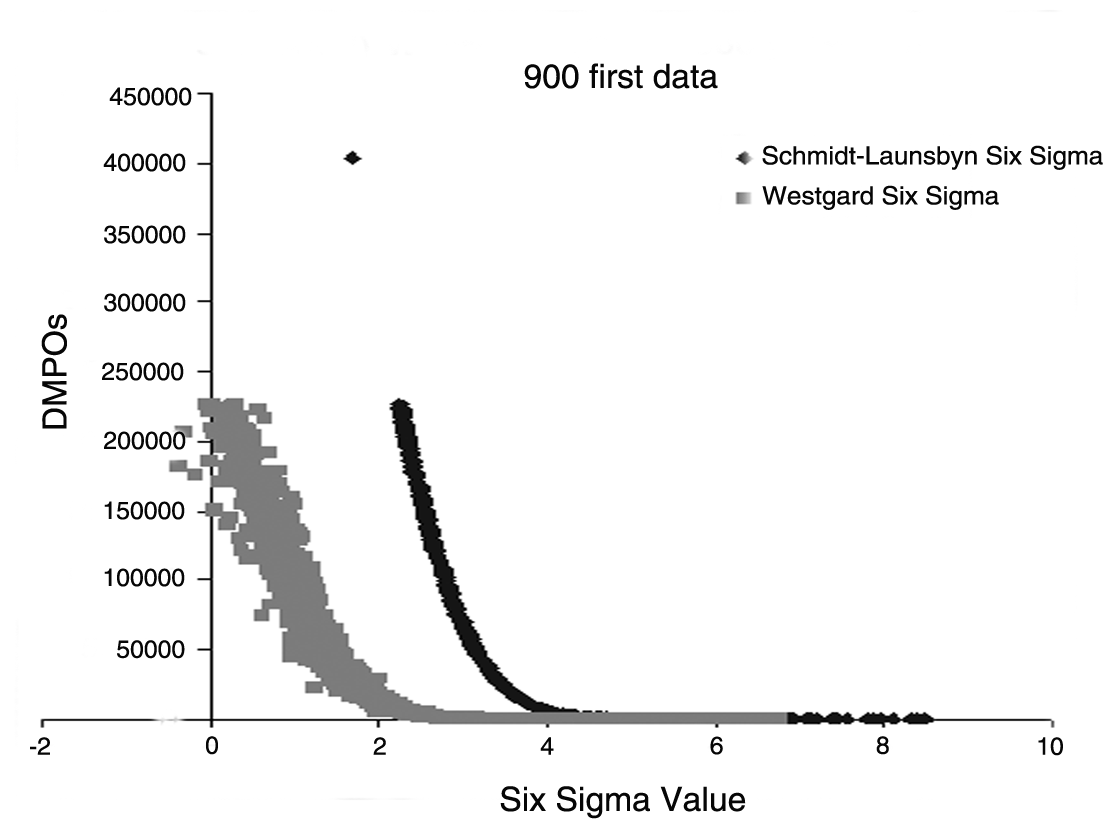

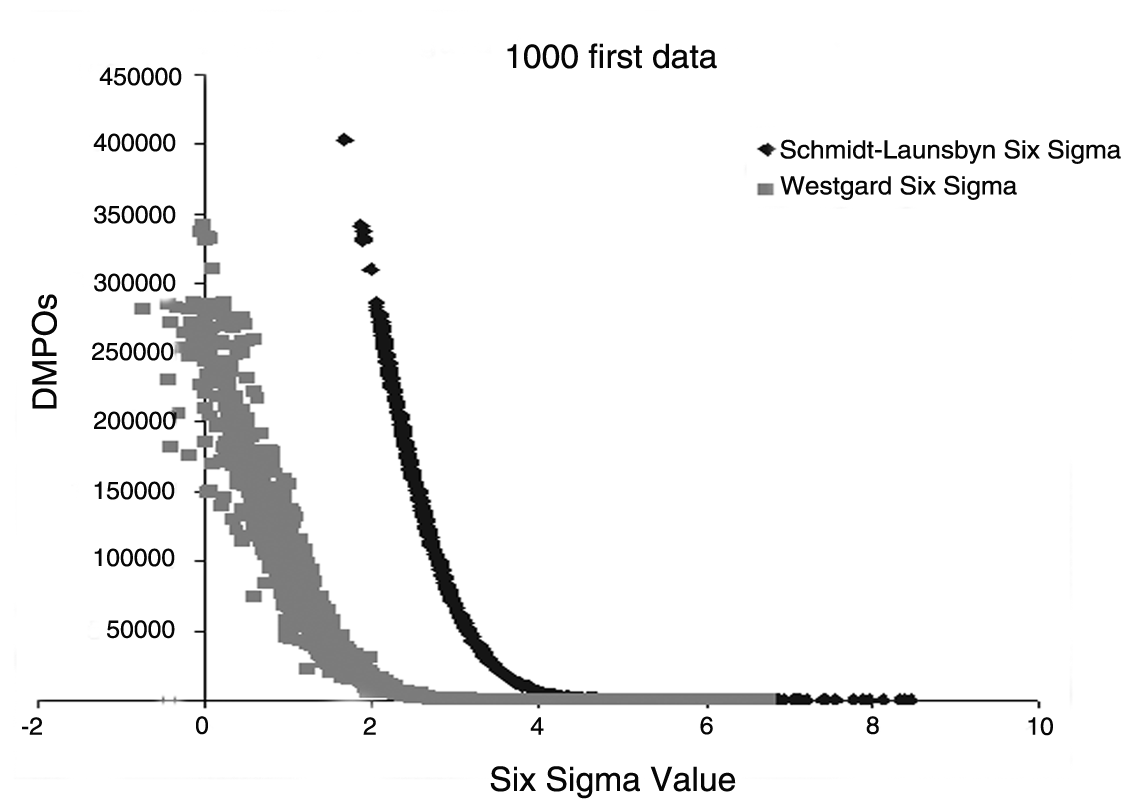
**

**
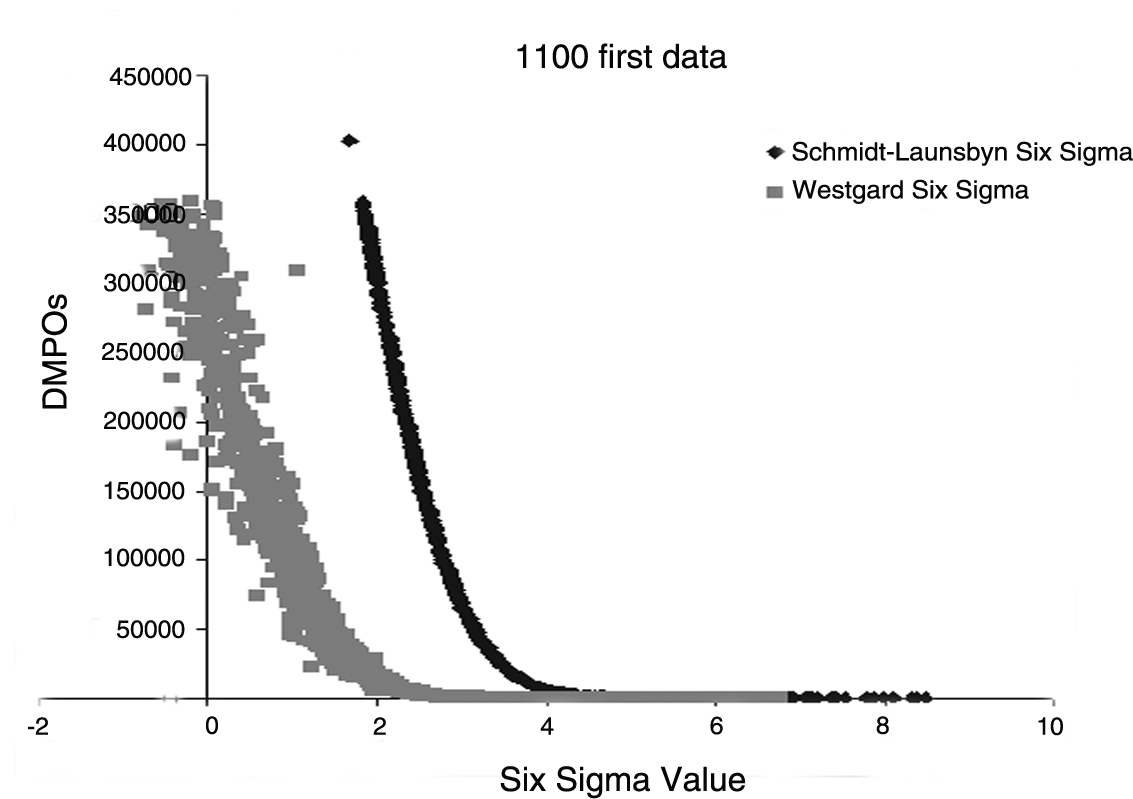

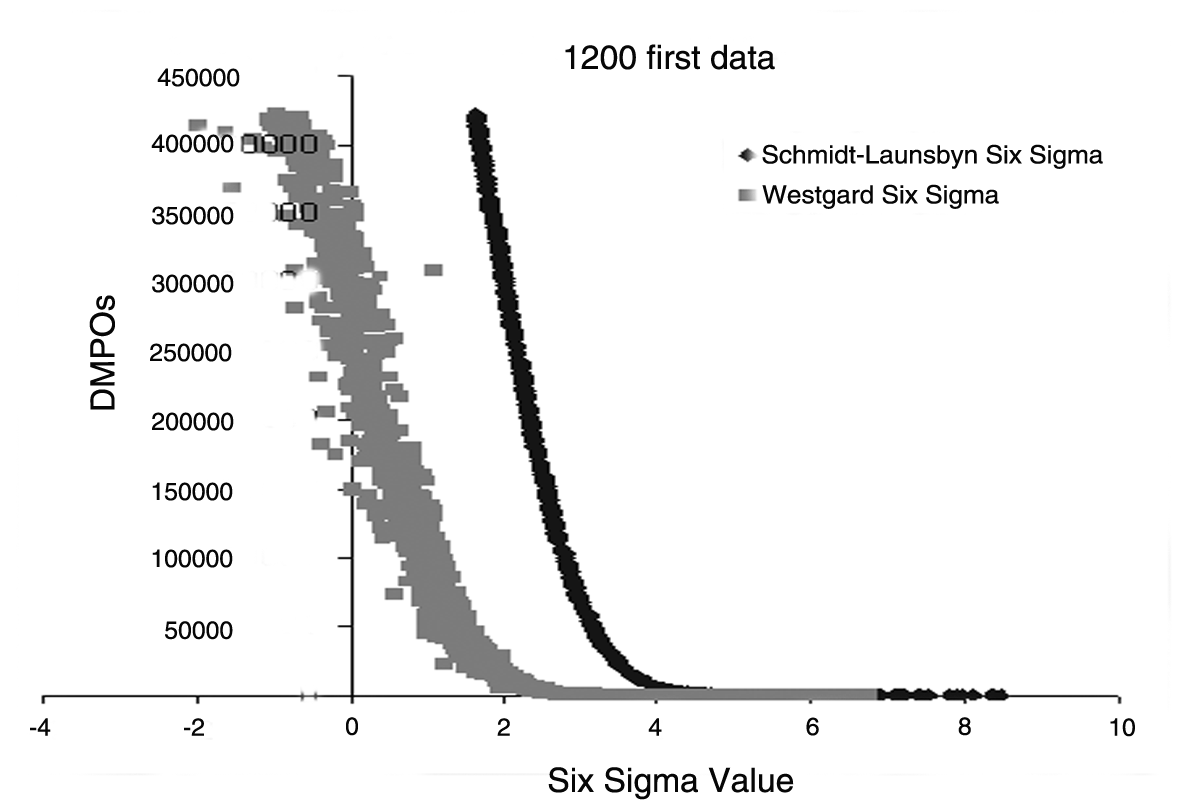
**

**Figura Suplementaria 2.** Relación entre el valor sigma calculado por la estrategia de Westgard (W) y por la estrategia Z-transformacion-ecuación Schmidt-Launsbyn (S-L)**.** (S-L)-W: Diferencia entre el cálculo de seis sigma por la estrategia de (S-L) y la estrategia W. Datos ordenados de menor a mayor valor de S-L, después de la eliminación de valores atípicos, al nivel de decisión clínica.

**
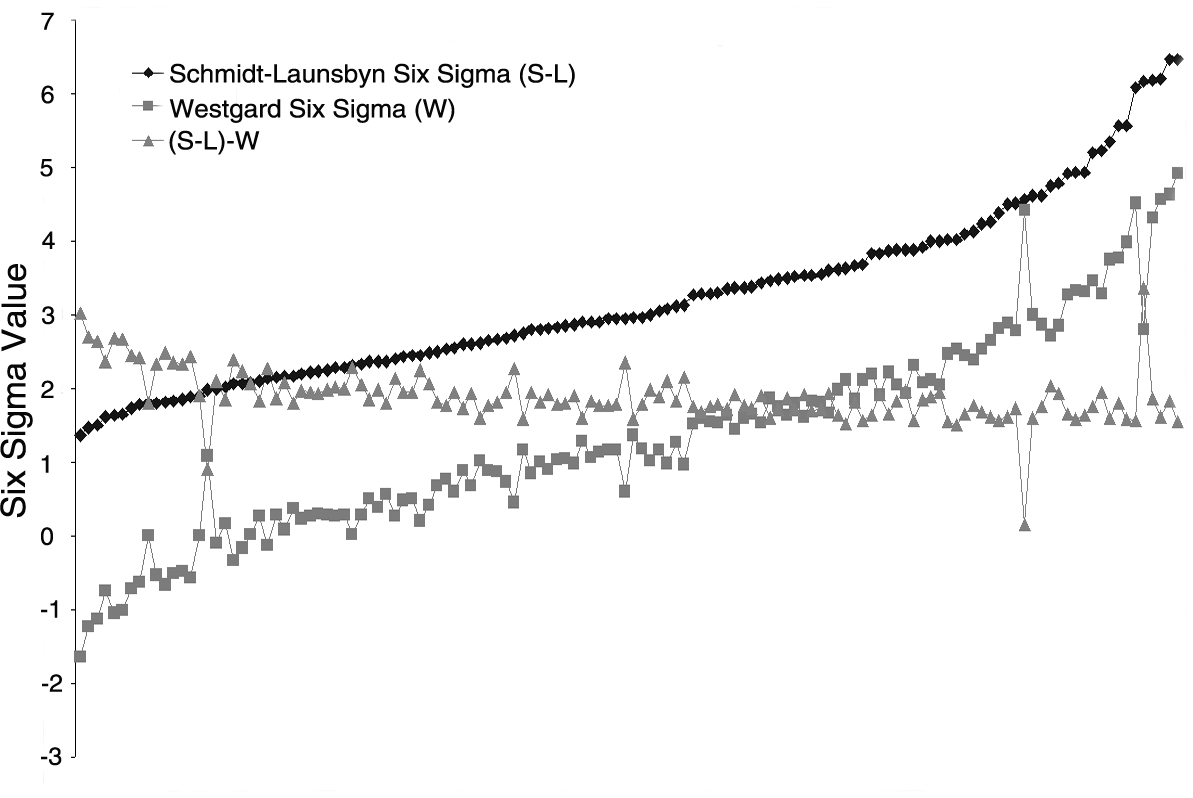
**

**Figura Suplementaria 3.** Relación entre el valor sigma calculado por la estrategia de Westgard (W) y por la estrategia Z-transformacion-ecuación Schmidt-Launsbyn (S-L)**.** (S-L)-W: Diferencia entre el cálculo de seis sigma por la estrategia de (S-L) y la estrategia W. Datos ordenados de menor a mayor valor de S-L, incluyendo los valores atípicos, al nivel de decisión clínica.

**
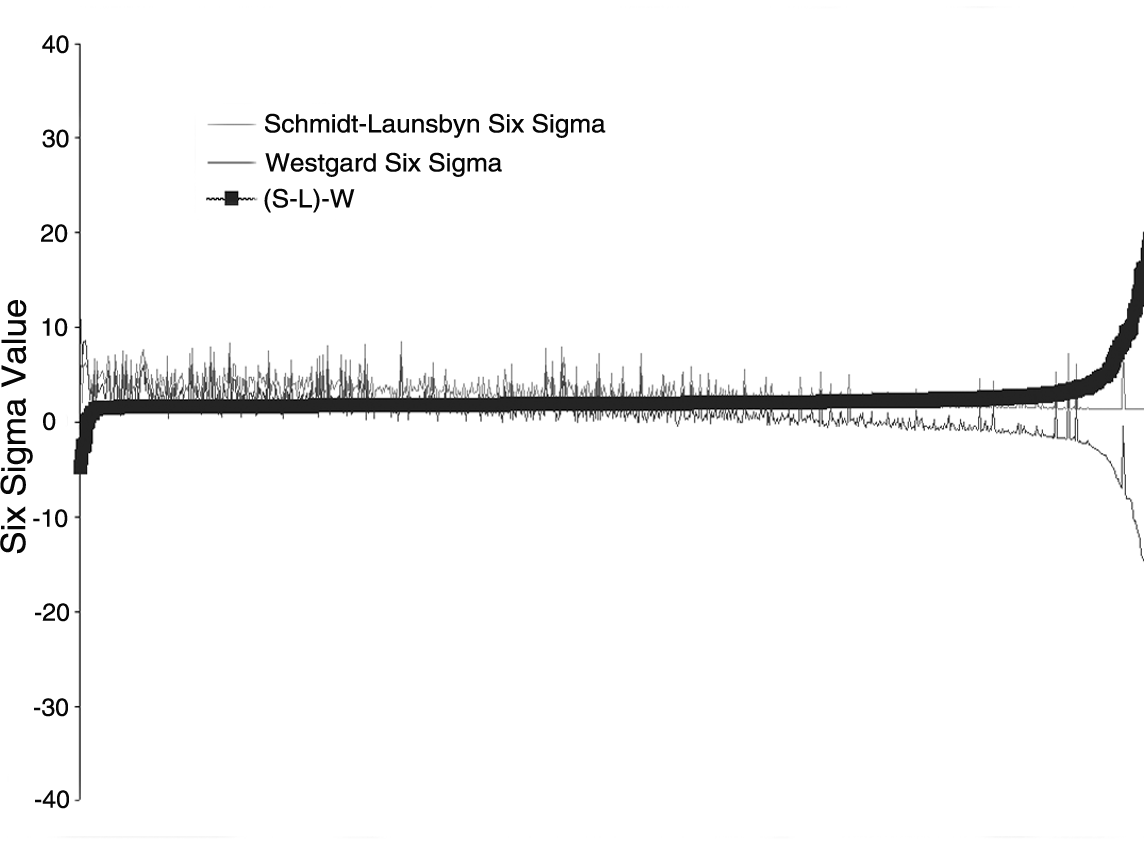
**

**Figura Suplementaria 4.** Relación entre el porcentaje del sesgo, el valor de la imprecisión y el ratio sesgo/imprecisión. En esta representación se incluyen los valores atípicos.

**
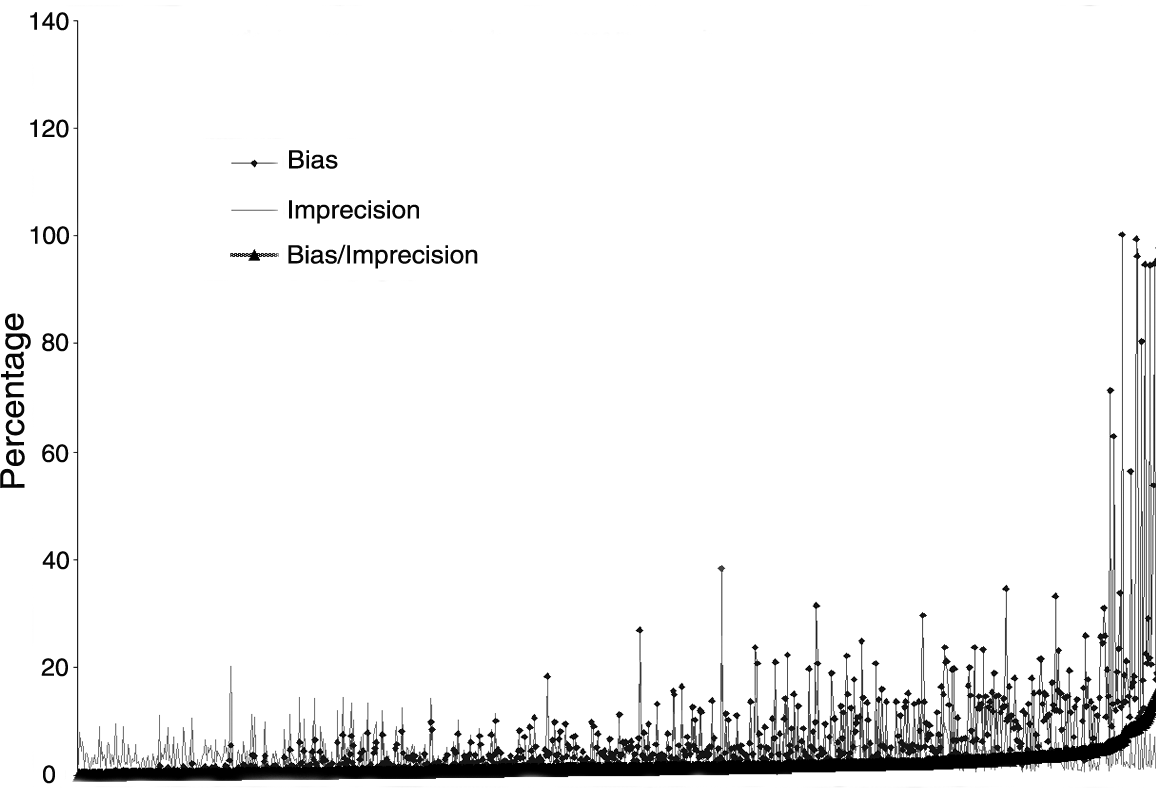
**
